# Impact of mother’s own milk *vs.* donor human milk on gut microbiota colonization in preterm infants: a systematic review

**DOI:** 10.20517/mrr.2024.44

**Published:** 2024-11-21

**Authors:** Jing Chen, Aranka J. van Wesemael, Nerissa P. Denswil, Hendrik J. Niemarkt, Johannes B. van Goudoever, Vanesa Muncan, Tim G.J. de Meij, Chris H.P. van den Akker

**Affiliations:** ^1^Department of Neonatology, Emma Children’s Hospital, Amsterdam UMC, University of Amsterdam, Amsterdam 1105 AZ, the Netherlands.; ^2^Amsterdam Reproduction and Development Research Institute, Amsterdam UMC, Amsterdam 1105 AZ, the Netherlands.; ^3^Amsterdam Gastroenterology Endocrinology Metabolism Research Institute, Amsterdam UMC, Amsterdam 1105 AZ, the Netherlands.; ^4^Medical Library, Amsterdam UMC, location University of Amsterdam, Amsterdam 1105 AZ, the Netherlands.; ^5^Department of Neonatology, Maxima Medical Centre, Veldhoven 5504 DB, the Netherlands.; ^6^Department of Electrical Engineering, Technical University Eindhoven, Eindhoven 5612 AZ, the Netherlands.; ^7^Department of Pediatric Gastroenterology, Emma Children’s Hospital, Amsterdam UMC, location University of Amsterdam, Amsterdam 1105 AZ, the Netherlands.; ^#^Authors contributed equally.

**Keywords:** Fecal microbiome, premature neonate, milk bank, donor milk, breastmilk, human milk

## Abstract

**Background:** Nutritional intake in preterm infants is associated with short- and long-term outcomes. The favorable outcomes of preterm infants who predominantly receive their mother’s own milk (MOM) are thought to be mediated partly through beneficial effects on the gut microbiome. When MOM is not available, donor human milk (DHM) is recommended as the best alternative. However, DHM is less effective in preventing adverse outcomes, which may be explained by compositional differences between MOM and DHM, resulting in different microbiome development. This systematic review focuses on the effects of predominant DHM *vs.* MOM feeding on the gut microbiota composition in preterm infants.

**Methods:** A comprehensive search was conducted across MEDLINE, Embase, and Cochrane databases. Eight out of the 717 publications identified were included. Data on gut microbiota composition, alpha diversity, and taxonomic differences between DHM- and MOM-fed preterm infants were extracted and analyzed.

**Results:** The microbiome composition was distinct between the two feeding groups. Alpha diversity measures were lower in DHM-fed infants, particularly when preterm formula (PF) was also provided. DHM-fed infants showed higher abundances of *Staphylococcaceae* and *Clostridiaceae*, and lower abundances of Bacteroidetes and *Bifidobacterium*.

**Conclusion:** The observed gut microbiome differences in DHM-fed preterm infants have previously been linked to adverse health outcomes. This underlines the importance of increasing the awareness of MOM intake in preterm infants. Further studies should explore the mechanisms through which human milk affects health outcomes.

## INTRODUCTION

Infants born very preterm (i.e., < 32 weeks of gestation) constitute a vulnerable population, characterized by a significant risk of mortality and short- and long-term morbidities^[1]^. Enteral feeding strategies are suggested to reduce those risks^[2]^. Enteral feeding with mother’s own milk (MOM) improves short- and long-term health outcomes compared to preterm formula (PF). This includes lower rates of necrotizing enterocolitis (NEC) and sepsis, and better neurodevelopmental outcomes^[3-7]^.

In the very preterm population, achieving an exclusive MOM diet is not always feasible^[8]^. Historically, when sufficient MOM was unavailable, very preterm infants received PF. In recent years, with the establishment of more donor human milk (DHM) banks, DHM is recommended as the second-best option by organizations such as the World Health Organization and the European Society for Paediatric Gastroenterology, Hepatology and Nutrition^[5,9-13]^. Albeit probably not to the same extent as MOM, DHM also leads to fewer adverse neonatal outcomes, such as NEC, as compared to PF^[7,14]^. Research comparing neonatal health outcomes between predominantly MOM or DHM diets is scarce and the available results are inconsistent^[7,15-17]^. Some studies indicate that the incidence of NEC and other morbidities may be dependent on the quantity of MOM provided to the infants, indicating that higher MOM exposition confers greater benefits to the very preterm infant compared to DHM^[18]^.

A potential mechanism through which human milk exerts its beneficial effects is via the composition and function of the gut microbiome^[19,20]^. Bioactive factors in MOM, such as lactoferrin, human milk oligosaccharides, secretory immunoglobulins, and the milk microbiota, influence the development of the preterm gut microbiota^[21]^. However, it is essential to recognize that some of these bioactive factors are affected by processing methods, including Holder pasteurization and multiple freeze-thaw cycles, to provide preterm recipients with a safe product of DHM. In addition, biological factors such as the lactation stage and characteristics of the donating mother may also influence the quality of the donated milk^[22]^. Subsequently, differences in composition between MOM and DHM may result in a difference in gut microbial colonization of preterm infants.

Currently, only a limited number of studies have compared the effect of DHM *vs.* MOM on the gut microbiota composition in preterm infants, hampering drawing a firm conclusion. This is important however, as clear associations between early-life gut microbiota composition and short- and long-term adverse outcomes, including NEC, sepsis, or neurodevelopmental impairment, have previously been described^[23-25]^.

The aim of this systematic review is to assess and provide a qualitative synthesis of available literature that reports on the effects of DHM *vs.* MOM on the gut microbiota composition in preterm infants, with specific attention to microbial diversity and taxonomic composition.

## METHODS

### Registration

This systematic review was registered at PROSPERO international prospective register of systematic reviews under number CRD42022358080. It was conducted according to the guidelines of the preferred reporting items for systematic review and meta-analysis protocols (PRISMA)^[26]^.

### Eligibility criteria

We included studies describing the fecal microbiota composition of preterm infants born < 37 weeks of gestation, with data available for at least two distinct groups of infants. One group received > 50% MOM, while the other group received > 50% DHM, at least until achieving full enteral feeding. Study cohorts in which infants had received predominantly formula feeding (defined as > 50% of total enteral volume) during this period were excluded. The use of human milk fortifiers with protein from bovine origin was not a criterion for exclusion. Studies using only conventional culturing methods were excluded, since this technique does not cover the entire gut microbiota composition^[27]^. Since molecular culture-independent techniques for gut microbiota analysis became available from 1990 onwards^[28]^, only studies published since then were eligible for inclusion. Given the ethical considerations and impossibilities of randomizing the provision of MOM, our literature search focused on observational studies and other relevant research designs.

### Information sources and search strategy

A comprehensive search was conducted in MEDLINE (Ovid), Cochrane Library, and EMBASE (Ovid) by J.C., A.v.W., and a medical information specialist (N.P.D.). Searches were restricted to articles published in English and focused on human subjects. The final search was completed on January 15th, 2024. Literature search strategies were developed using a combination of controlled terms and titles and abstract words related to the concepts: preterm infants, enteral feeding, human milk, DHM, and gut microbiota. The search terms were combined using Boolean operators AND and OR. No data limit was applied. Duplicates were removed with DedupEndNote (version 1.0.1)^[29]^. The full search strategy is included in Supplementary File 1. In addition, the International Clinical Trials Registry Platform Search Portal and ClinicalTrials.gov were searched for ongoing or recently completed trials and PROSPERO was searched for ongoing or recently completed systematic reviews. Backward snowballing techniques were used to identify potentially relevant articles from the references of included articles.

### Study selection and data extraction

Two authors (J.C and A.v.W) independently screened the titles and abstracts retrieved from the search against the predefined inclusion and exclusion criteria. Full reports for all titles that appeared to meet the inclusion criteria or where there was any uncertainty were obtained. Review author pairs screened the full-text reports and decided whether these met the inclusion criteria. We recorded the reasons for excluding trials.

Standardized forms were used to extract data from the original studies by J.C. and A.v.W. Inconsistencies were resolved by discussion with T.G.M. and C.H.P.A. or study authors were contacted if there were important uncertainties. We extracted the following data: primary author, year of publication, geographic location of the study, demographic data, duration of study, proportion of predominant feeding type (MOM or DHM), number of participants, time points of fecal sample collection, total numbers of samples analyzed, and microbiota analysis method. The main outcome data that were extracted included alpha and beta diversity and microbial composition at different taxonomic levels. When reported, clinical outcomes and other fecal analysis results were extracted as they might provide relevant insights into the mechanistic effects the microbiome differences could have on health outcomes.

### Outcomes measures and data presentation

To acquire a robust assessment of the impact of feeding MOM or DHM on intestinal microbiota, and to mitigate potential bias caused by the effects of any consumption of PF, two subgroups were delineated based on the infants’ feeding regimen from birth till full enteral feeding achieved, or up to the postmenstrual age (PMA) of 36 weeks:

● Subgroup A: studies where infants received 100% human milk (either MOM or DHM) ○ MOM group: received > 50% MOM ○ DHM group: received > 50% DHM

● Subgroup B: studies where infants received a combination of human milk (MOM or DHM) and PF ○ MOM group: received > 50% MOM (with < 50% DHM and/or PF) ○ DHM group: received > 50% DHM (with < 50% MOM and/or PF)

The MOM group serves as the reference group in all text, tables, and figures, unless stated otherwise.

### Risk of bias and quality assessment

The risk of bias in longitudinal studies was assessed using the Newcastle-Ottawa Scale (NOS) for observational studies^[30]^. This instrument measures four domains: participant selection, comparability, exposure, and outcome. It can be modified to better fit the specific subject of interest. The NOS used for this review was modified by the authors to suit this review and is found in Supplementary File 2. The scoring is based on the number of stars, with longitudinal studies being eligible for a maximum score of nine stars.

### Data synthesis

Due to the expected heterogeneity of study designs and outcomes, we did not aim to provide a meta-analysis.

## RESULTS

### Included studies

**Figure 1:**
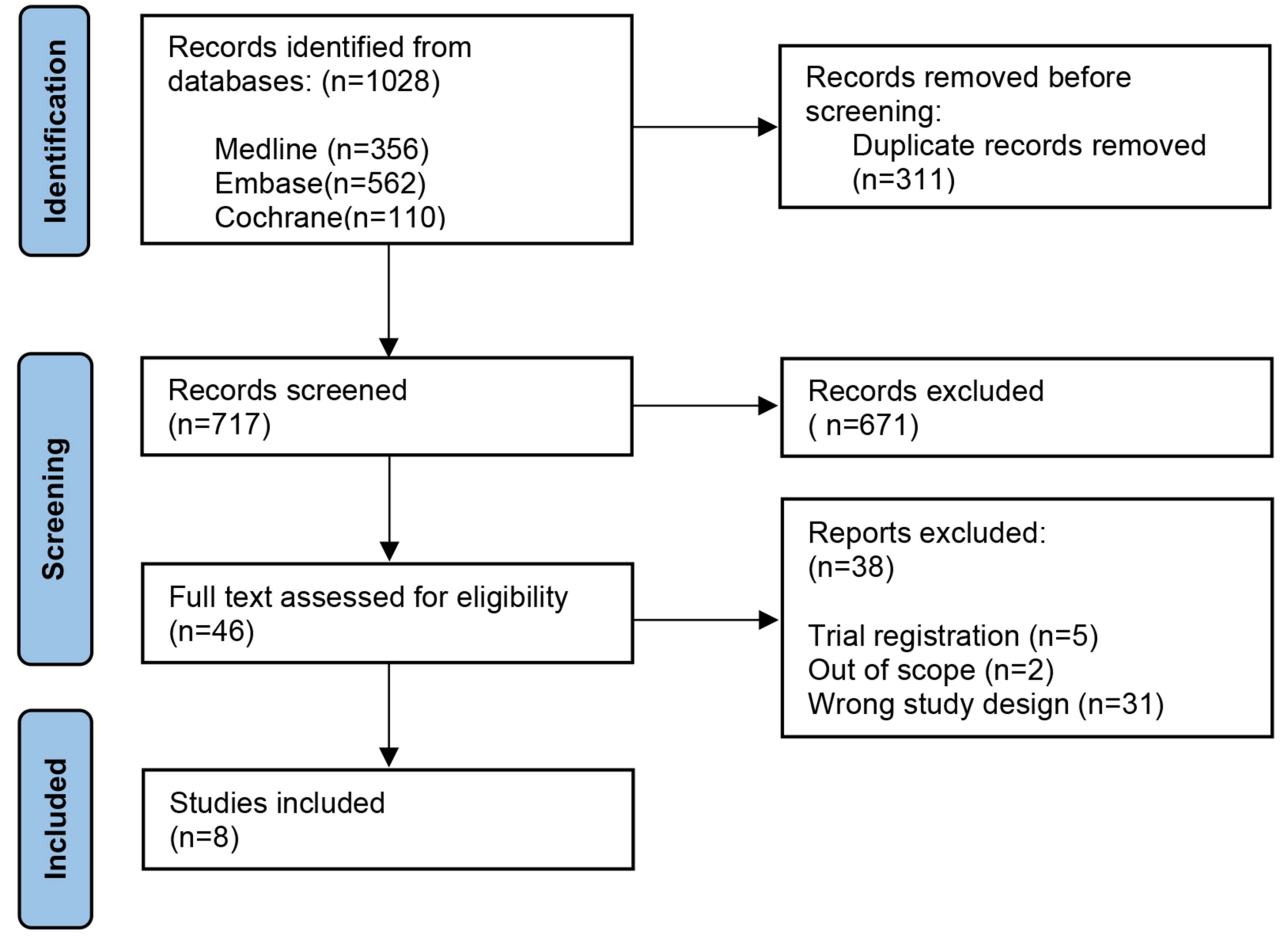
PRISMA 2020 flow diagram of the search and selection process. PRISMA: Preferred reporting items for systematic review and meta-analysis protocols.

The search process is shown in Figure 1. A total of 1,028 articles were identified through the database search. After removing duplications, 717 articles were initially screened by title and abstract, of which 671 articles could be excluded. In total, 46 articles underwent full-text review, of which 38 articles were excluded. Thus, a total of 8 original studies could be included in our qualitative synthesis^[19,31-37]^. Backward snowballing did not yield any additional suitable articles.

### Description of the studies

**Figure 2:**
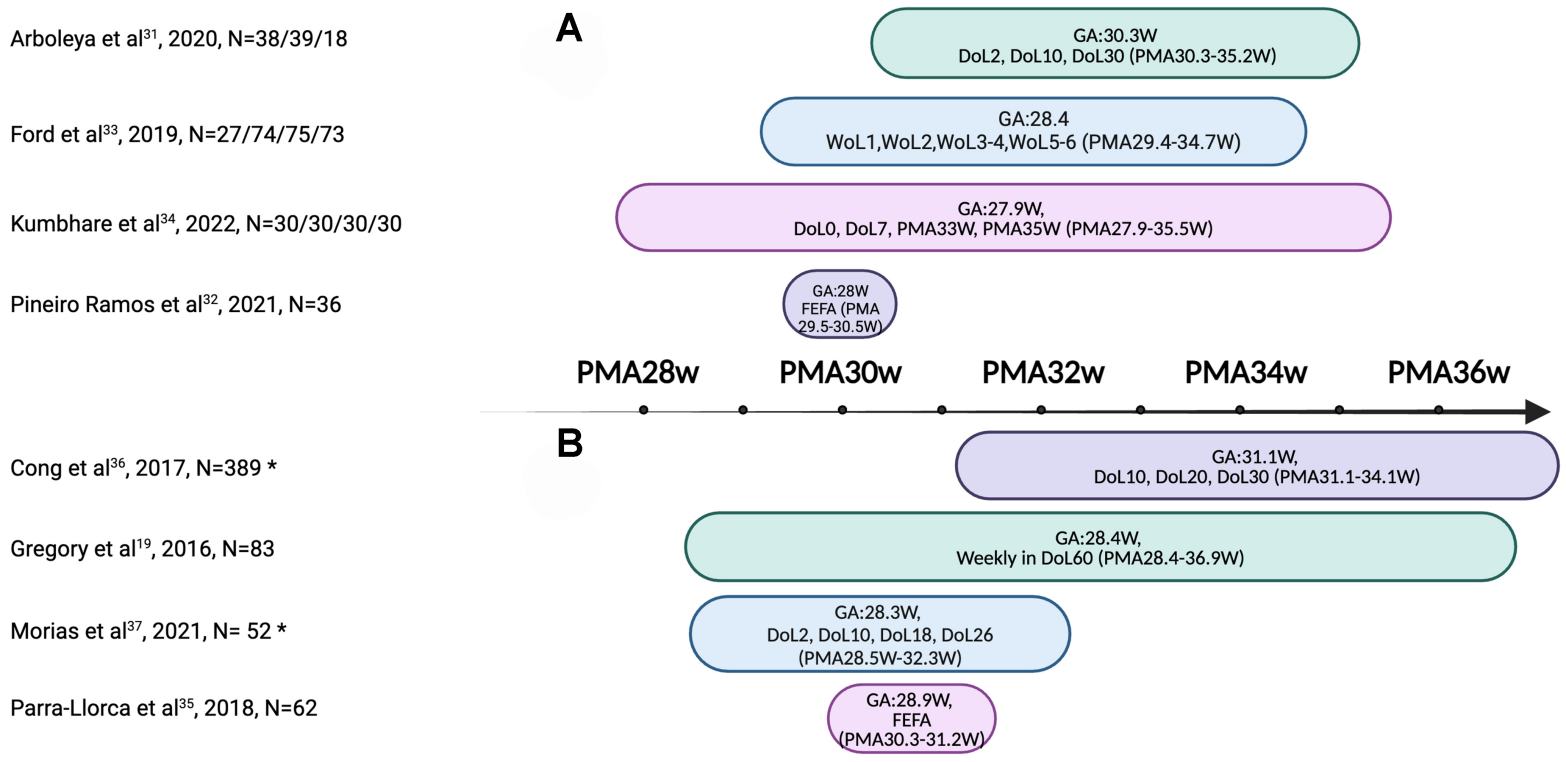
Overview of studies included in this review. On the X-axis, the sampling period is shown, expressed in weeks of PMA. (A) Subgroup A consists of studies where infants received 100% human milk (MOM/DHM); (B) Subgroup B consists of studies where infants received a combination of human milk (MOM/DHM) and PF. Note: To express the maturity stage of infants at the time of sample collection, we calculated PMA during sampling collection time points. For the start of the PMA range, we added the earliest time point of sample collection to the lowest mean or median gestational age from both groups (MOM and DHM). For the end of the PMA range, we added the latest time point of sample collection to the highest mean or median gestational age from both groups. For studies that stated sampling at the day of achieving full enteral feeding, but not explicitly mentioning at which day of life this was, we arbitrarily chose ten days of age as the mean age of achieving full enteral feeding in order to calculate the PMA during sample collection^[38]^. N means number of stool samples collected at one or multiple time points. ^*^N for all time points combined. Created with Biorender.com. PMA: Postmenstrual age; MOM: mother’s own milk; DHM: donor human milk; PF: preterm formula; GA: gestational age; DoL: day of life; WoL: week of life; FEFA: full enteral feeding achieved.

Characteristics of the eight included studies are summarized in Table 1 and depicted in Figure 2. Seven out of the eight studies were prospective observational cohorts. The remaining study by Kumbhare *et al.* was a randomized, controlled, open-label trial comparing bovine milk-based *vs.* human milk-based fortifier added to MOM or, if insufficiently available, to DHM^[34]^. However, the authors also described fecal microbiota results for two post-hoc cohorts where infants were stratified whether they had predominantly received MOM or DHM, irrespective of the fortifier allocation.

**Table 1:** Characteristics of studies included in this review

**Author, year, country [ref]**	**Design**	**Primary aim**	**Study population**	Number of infants^*^ (MOM/DHM)	**Baseline characteristics MOM group**	**Baseline characteristics DHM group**	**Subgroup and definition of feeding groups**
**Subgroup A**							
Arboleya *et al.*, 2020, Spain^[31]^	Mono-center cohort study	Effect of DHM on Bifidobacteria	GA < 34 weeks	42 (in total)	GA: 30.9 (2.44) BW: 1,351 (353) MoD: 46% vaginal	GA: 30.3 (2.7) BW: 1,246 (302) MoD: 42% vaginal	A MOM: 100% DHM: 100%
Ford *et al.*, 2019, USA^[33]^	Mono-center cohort study	Effect of human milk on microbiota development	BW < 1,500 grams	90 (58/32)	GA: 28.7 (2.0) BW: 1,107 (242) MoD: 18% vaginal	GA: 28.4 (2.5) BW: 1,066 (294) MoD: 12% vaginal	A MOM: > 50% DHM: > 50%
Kumbhare *et al.*, 2022, Canada^[34]^	Stratified cohorts from a mono-center randomized controlled open-label trial	Gut microbiome, oxidative stress and inflammation in milk fortifiers	GA 26-30 weeks	30 (20/10)	GA: 27.9 (1.0) BW: 1,013 (159) MoD: 20% vaginal	GA: 28.4 (0.86) BW: 1,053 (159) MoD: 20% vaginal	A MOM: > 50% DHM: > 50%
Piñeiro-Ramos *et al.*, 2020, Spain^[32]^	Mono-center cohort study	Composition of microbiota and urinary metabolites	GA < 32 weeks and/or BW < 1,500 grams	36 (18/18)	GA: 28 (27-29) BW: 1,300 (300) MoD: 45% vaginal	GA: 29 (28-30) BW: 1,400 (200) MoD: 45% vaginal	A MOM: 80% DHM: 80%
**Subgroup B**							
Cong *et al.*, 2017, USA^[36]^	Bi-center cohort study	Effect of feeding type on gut microbial patterns	GA 28 to 32 + 6 weeks	18 (15/3)	GA: 31.1 (1.8) BW: 1,444.2 (442.7) MoD: 40% vaginal^**^		B MOM: > 70% DHM: > 70%
Gregory *et al.*, 2016, USA^[19]^	Mono-center cohort study	The influence of nutrition on the microbiome	GA < 32 weeks	20 (10/10)	GA: 28.4 (1.5) BW: 1,044 (258) MoD: 10% vaginal	GA: 28.4 (2.1) BW: 1,070 (422) MoD: 10% vaginal	B MOM: > 50% DHM: > 50%
Morais *et al.*, 2021, Portugal^[37]^	Mono-center cohort study	Impact of different feeding types on fecal microbiota and ALP activity	GA < 32 weeks	95 (75/20)	GA: 28.3 (2.1) BW: 1,123 (345) MoD: 45% vaginal	GA: 28.6 (1.8) BW: 1,173 (284) MoD: 35% vaginal	B MOM: > 50% DHM: > 50%
Parra-Llorca *et al.*, 2018, Spain^[35]^	Mono-center cohort study	Effect of nutrition on gut microbiota composition	GA < 32 weeks and/or BW < 1,500 grams	62 (34/28)	GA: 28.9 (1.9) BW: 1,228 (301) MoD: 59% vaginal	GA: 29.8 (2.4) BW: 1,304 (262) MoD: 40% vaginal	B MOM: > 80% DHM: > 80%

^*^In groups of interest; ^**^for all included infants, not stated separately for DHM or MOM group. MOM: Mother’s own milk; DHM: donor human milk; GA: gestational age (in weeks), expressed as mean (SD) or median (IQR); BW: birthweight (in gram), expressed as mean (SD) or median (IQR); MoD: mode of delivery, expressed as percentages; ALP: alkaline phosphates.

The criteria that were used to stratify infants in one of the two feeding groups varied among the studies. Studies used varying thresholds from a minimum of 50%^[33,34,37]^ to 70%^[36]^, 80%^[32,35]^, or by exclusively receiving 100% MOM or 100% DHM^[31]^ of the total milk volume intake. In the study by Gregory *et al.*, infants were also stratified based on whether they had received exclusively MOM, while those in the DHM group switched from exclusively DHM (duration of 6 to 25 days) to PF once achieving full enteral feeding^[19]^. Eventually, four studies were grouped into Subgroup A^[31-34]^, and Subgroup B consisted of the other four^[19,35-37]^. Six studies excluded children with NEC and/or sepsis^[19,31,34-37]^. Ford *et al.* reported an overall NEC rate of 2.4% among the study infants^[33]^. Piñeiro-Ramos *et al.* reported only one NEC case (5%) in the DHM group^[32]^.

A total of 389 infants were included, with analyses conducted on 1,832 unique fecal samples collected from the second day of life up to the 90th day of life. The mean/median gestational age in the included cohorts ranged from 27.9 to 31.1 weeks, and the mean/median birth weights ranged from 1,013 to 1,444 grams. Cesarean section was the predominant mode of delivery in all but one study^[35]^. Overall, there were no major clinically meaningful differences in reported baseline characteristics between infants in the MOM and DHM cohorts in each of the included studies.

Two studies used a cross-sectional approach, with one sample per individual^[32,35]^ collected at the time of full enteral feeding achieved, while the remaining six studies collected multiple samples at different time points during admission.

The applied gut microbiota analysis techniques in each study are listed in Supplementary Table 1. Next-generation sequencing was employed in all eight studies to measure specific 16S rRNA gene regions. In the study by Arboleya *et al.*, the profiling is specific to the species composition of the genus *Bifidobacterium* only, whereas others studied overall gut microbiota composition^[31]^.

### Risk of bias and quality of the evidence

The assessment of the included cohort studies using the NOS is shown in Table 2*.* Four studies^[31,33,34,37]^ had a maximum score of 9 stars, two studies^[19,36]^ 8 stars, and two studies^[32,35]^ 7 stars. The representativeness of the DHM cohort in the study by Cong *et al.* was limited^[36]^, because there were only two or three infants at each observed time point (the average number of stool collections in this study for each infant was 12.7). Piñeiro-Ramos *et al.* and Parra-Llorca *et al.* only collected stool samples once when the infants reached full enteral feeding, so those studies scored 0 on “long-enough follow-up” or “adequacy of follow-up cohort”^[32,35]^. The study by Gregory *et al.* was characterized by a high risk of bias due to varied DHM exposure levels given the switch to PF at various time points per infant (8 stars)^[19]^.

**Table 2:** Quality assessment using the adapted NOS of all included articles

**Author, year**	**Selection 1**	**Selection 2**	**Selection 3**	**Selection 4**	**Comparability 1**	**Outcome 1**	**Outcome 2**	**Outcome 3**	**Total**
Arboleya *et al.*, 2020^[31]^	B^*^	A^*^	A^*^	A^*^	A^**^	B^*^	A^*^	A^*^	9
Cong *et al.*, 2017^[36]^	E	A^*^	A^*^	A^*^	A^**^	B^*^	A^*^	A^*^	8
Ford *et al.*, 2019^[33]^	A^*^	A^*^	A^*^	A^*^	A^**^	B^*^	A^*^	A^*^	9
Gregory *et al.*, 2016^[19]^	B^*^	A^*^	A^*^	A^*^	B^*^	B^*^	A^*^	A^*^	8
Khumbare *et al.*, 2022^[34]^	A^*^	A^*^	A^*^	A^*^	A^**^	B^*^	A^*^	A^*^	9
Morais *et al.*, 2021^[37]^	A^*^	A^*^	A^*^	A^*^	A^**^	B^*^	A^*^	A^*^	9
Parra-Llorca *et al.*, 2018^[35]^	B^*^	A^*^	A^*^	A^*^	A^**^	B^*^	no FU	n.a.	7
Piñeiro-Ramos *et al.*, 2021^[32]^	B^*^	A^*^	A^*^	A^*^	A^**^	B^*^	no FU	n.a.	7

Scoring by means of letters and stars are further explained in Supplementary File 2. Maximum score is 9 stars. A study can be awarded a maximum of one star for each numbered item within the selection and outcome categories. A maximum of two stars can be given for comparability. NOS: Newcastle-Ottawa Scale; FU: follow-up; n.a.: not applicable.

### Gut microbiome outcomes

The outcomes of this part are described under the following items: (1) Alpha diversity (a measure of microbiome diversity within a single sample), (2) Beta diversity (a measure of similarity of microbiota composition between groups), (3) taxonomy, (4) fecal metabolites, and (5) clinical outcomes. The MOM group serves as a reference group in text, tables, and figures, unless stated otherwise.

### Alpha diversity

#### Subgroup A

Table 3 provides an overview of all fecal microbiota outcomes***.*** Three out of four studies reported findings on alpha diversity from the second day of life to the sixth week of life^[31-33]^. Arboleya *et al.* reported a higher Shannon index from day 10 onwards, with the Chao1 index only higher at day 10 in the DHM group compared to MOM (all *P*-values < 0.05)^[31]^. In addition, they reported a decreasing alpha diversity over time in the MOM group, but the decrease was less pronounced in the DHM group. It is important to note that in the study by Arboleya *et al.*, these diversity measures are specific to *Bifidobacterium* bacteria only, rather than overall diversity^[31]^. The remaining two studies showed no difference in alpha diversity indices, but Ford *et al.* observed lower operational taxonomic unit (OTU) richness in the DHM group across all time points (*P* = 0.013)^[33]^.

**Table 3:** Fecal microbiota outcomes of all included studies

**Author, year, country**	**Time points**	**Amount of samples**	**Alpha diversity**	**Beta diversity**	**Taxonomy (phylum, class, order, family, genus, species)**	**Additional information**
**Subgroup A**						
Arboleya *et al.*, 2020, Spain^[31]^	1. DoL 2 2. DoL 10 3. DoL 30 4. DoL 90	141	T1: no significant difference T2: higher Shannon & Chao1 (*P* < 0.05) T3: higher Shannon (*P* < 0.01)	T2-3: redundancy analysis shows distinct separation (*P* = 0.028, *P* = 0.026, respectively)	T1: (species level) ↑ *B. longum spp. Suis* (*P* < 0.05) T2: (species level) ↑ *B. animalis spp. Lactis*, *B. longum spp. Suis*, *B. bifidum*, *B. pseudolongum spp pseudolongum* (all *P* < 0.05) ↓ *B. longum spp longum*, *B. vansindernii*, *B. reuteri* (*P* < 0.05) T3: (species level) ↑ *B. bifidum*, *B. dentium*, *B. animalis spp lactis*, *B. magnum* (all *P* < 0.05)	*Bifidobacterium* diversity measured, not overall diversity
Ford *et al.*, 2019, USA^[33]^	1. WoL 1 2. WoL 2 3. WoL 4 4. WoL 6	546	T1-4: ↓ OTU richness (*P* = 0.013) Similar Shannon index Increasing alpha diversity over time	There was no distinct microbial clustering per week of life in the DHM group, whereas this was present in the MOM group	T1-2: (phylum level) no significant differences T3: (phylum level) ↓ Actinobacteria, ↑ Firmicutes (both *P* < 0.05) (Genus level) ↑ *Staphylococcus* (*P* = 0.014), ↓ *Bacteroides*, *Bifidobacterium*, *Enterococcus* (all *P* < 0.05)	
Kumbhare *et al.*, 2022, Canada^[34]^	1. Day 0 2. Day 7 3. PMA 33 weeks 4. PMA 35 weeks	112	Not stated	T3-4: MOM intake explained 22% and 18% of Bray-Curtis dissimilarity (both *P* < 0.01)	All time points: (Genus level) ↑ *Clostridium*, unclassified Lactobacilliales (all *P* < 0.001), ↓ *Propionibacterium*, *Veillonella*, unclassified Enterobacteriaceae T4: (genus level) ↑ unclassified *Lactobacilliales*, ↓ *Bifidobacterium* (both *P* < 0.05)	Time points correspond with DoL 3-5 depending on fortification start
Piñeiro-Ramos *et al.*, 2020, Spain^[32]^	Full-enteral feeding achieved	36	No difference in Shannon or Chao1 index	PCoA (UNIFRAC) and PERMANOVA show microbial composition differences (*P* = 0.04)	(Family level) ↑ Staphylococcaceae, Pasteurellaceae (*P* < 0.05)	
**Subgroup B**						
Cong *et al.*, 2017, USA^[36]^	1. DoL 0-10 2. DoL 11-20 3. DoL 21-30	389^***^	T1-3: ↓ Gini-Simpson index (explained by feeding type, *P* < 0.01)	Feeding type explained 11% of Bray-Curtis dissimilarity (*P* < 0.01, PERMANOVA)	T1-3: (order level) ↑ Enterobacteriales, ↓ Clostridiales, Lactobacillales, Pasteurellales, Bacillales, Bifidobacteriales, Actinomycetales	
Gregory *et al.*, 2016, USA^[19]^	Daily until discharge or DoL 60	199^***^	↓ Shannon index; increased more rapidly over time	Feeding type explained 21% of the variance in Bray-Curtis dissimilarity (*P* < 0.001)^*^	(order level) ↑ Bacillales, Lactobacillales, succession of ↑ Enterobacteriales, Clostridiales, Bifidobacteriales with increasing PMA in both groups	
Morais *et al.*, 2021, Portugal^[37]^	1. DoL 2 2. DoL 8 3. DoL 16 4. DoL 26	389^***^ 61 for 16S rRNA sequencing	T4: ↓ Chao1 index (*P* < 0.05) No differences in Shannon index	T4: no beta diversity difference (Bray-Curtis PCoA)	RT-PCR T4: (phylum level) ↓ Firmicutes (*P* = 0.05)^**^ (Genus level) ↓ *Bifidobacterium* (adjusted for gestational age, *P* = 0.003; not significant in multivariable model) (species level) ↓ *E.Coli* (*P* < 0.05) 16S rRNA T4: (phylum level) ↑ Firmicutes, Proteobacteria, Bacteroidetes, Actinobacteria (Genus level) ↑ *Staphylococcus*, *Streptococcus* ↓ *Serratia*, *Clostridium*, *Bifidobacterium*	RT-PCR method measured key bacterial phyla and genera
Parra-Llorca *et al.*, 2018, Spain^[35]^	Full-enteral feeding achieved	62^***^	Not stated	RDA indicates microbial composition differences based on observed OTUs (*P* = 0.001)	(Phylum level) ↑ Firmicutes ↓ Actinobacteria (both *P* < 0.05) (Family level) ↑ Clostridiaceae ↓ Bifidobacteriaceae (both *P* < 0.05) (Genus Level) ↓ *Bifidobacterium*, unclassified Enterobacteriaceae (both *P* < 0.05) DESeq2: ↑ *Staphylococcus*, *Clostridium*, *Serratia*, *Coprococcus*, *Aggregatibacter*, *Lactobacillus*, ↓ *Bacteroides*, *Acinetobacter*, *Haemophilus* (all *P* < 0.05)	

^*^This includes a formula feeding group; ^**^model adjusted for: gestational age, mode of delivery, z-scores growth parameters at birth, infant’s antibiotic therapy received within 8 days prior to fecal collection; ^***^total number of samples, unknown number for subgroups. DoL: Day of life; WoL: week of life; OTU: operational taxonomic unit; DHM: donor human milk; MOM: mother’s own milk; PMA: postmenstrual age; PCoA: principal coordinates analysis; UNIFRAC: unique fraction metric; PERMANOVA: permutational multivariate analysis of variance; RT-PCR: reverse transcription polymerase chain reaction; RDA: redundancy analysis.

#### Subgroup B

Three out of four studies reported findings on alpha diversity from birth to day 60 of life^[19,36,37]^. In all three studies, alpha diversity measures were found to be lower in the DHM group compared to the MOM group. Cong *et al.* observed that the Gini-Simpson index increased over time in both groups, but was continuously lower in the DHM group from birth to the 30th day of life, with feeding type explaining this difference (*P* < 0.01)^[36]^. Gregory *et al.* showed a lower Shannon diversity index overall, although it increased more rapidly over time compared to the MOM group^[19]^. Morais *et al.* showed a lower Chao1 index on the 26th day of life, but no differences in the Shannon index^[37]^.

#### Overall

Most studies reporting on different alpha diversity measures found either no difference or significantly lower alpha diversity in the DHM groups compared to those infants receiving predominantly MOM^[19,33,36,37]^. In contrast to the other studies, Arboleya *et al.* focused specifically on *Bifidobacterium* diversity solely, showing a higher Shannon diversity index in the DHM group at all time points starting from the tenth day of life, rather than reporting on overall microbial diversity^[31]^.

### Beta diversity

#### Subgroup A

All four studies reported beta diversity differences based on feeding type. Three studies demonstrated distinct clustering based on feeding type^[31,32,34]^. Arboleya *et al.* showed distinct separation from the tenth day of life onwards (*P* < 0.05)^[31]^. Kumbhare *et al.* showed that MOM intake explained 22% and 18% of the Bray-Curtis dissimilarity (*P* < 0.01) at PMA of 33 and 35 weeks, respectively^[34]^. Piñeiro-Ramos *et al.* showed a different microbial composition on the 10th and the 30th day of life (*P* = 0.04)^[32]^. Ford *et al.* observed different microbiome successions per week of life, with MOM-fed infants showing apparent clustering per week of life, whereas the DHM-fed infants did not^[33]^.

#### Subgroup B

All four studies reported findings on beta diversity. Three out of four studies showed distinct clustering based on feeding type^[19,35,36]^. Cong *et al.* and Gregory *et al.* showed feeding type explained the greatest variance in Bray-Curtis dissimilarity (11%, *P* < 0.01 and 21%, *P* < 0.001, respectively)^[19,36]^. Parra-Llorca *et al.* observed differences in microbial composition through multivariate redundant discriminate analysis^[35]^. Morais *et al.* assessed beta diversity only at postnatal day 26 (PMA around 34 weeks) but found no differences between feeding groups^[37]^.

#### Overall

All but one study reported findings on beta diversity between the DHM and MOM groups. Six of them showed distinct microbial clustering between DHM and MOM groups, starting from the 10th day of life^[19,31,32,34-36]^ until PMA 36 weeks. Morais *et al.* was the exception, reporting no differences^[37]^.

### Taxonomy

**Figure 3:**
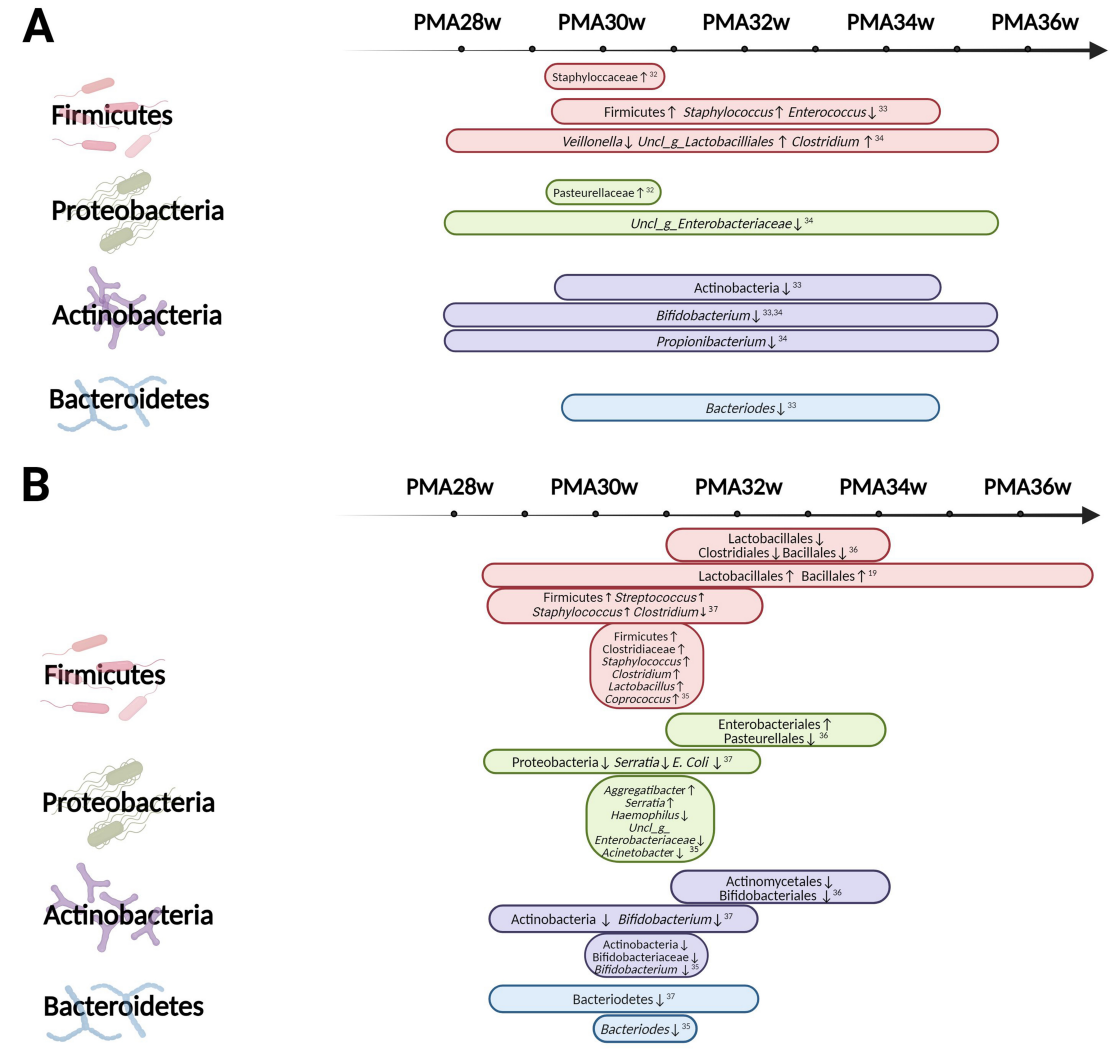
Graphical depiction of taxonomic differences at various PMA intervals. (A) subgroup A consists of studies in which infants received 100% human milk (MOM/DHM). Arboleya *et al.* were excluded from this figure because they only reported differences within the *Bifidobacterium* genus^[31]^; (B) subgroup B consists of studies in which infants received a combination of human milk (MOM/DHM) and PF. Created with Biorender.com. This figure is a visual complement to Table 3, where taxonomic differences resulting from DHM consumption in comparison to MOM at each time point are described. PMA: Postmenstrual age; MOM: mother’s own milk; DHM: donor human milk; PF: preterm formula.

The primary taxonomy differences between groups from birth till PMA 36 weeks are summarized in Table 3 and graphically depicted in Figure 3. Below are recurring differences mentioned across multiple studies, categorized by phylum, to illustrate commonalities in this heterogeneous section. The MOM group serves as a reference group in text, tables, and figures. No consistent differences at the taxonomic level were apparent between subgroup A and subgroup B. Therefore, no distinction between the subgroups was made in the text.

#### Firmicutes

Seven studies reported differences in the Firmicutes phylum at different taxonomic levels^[19,32-37]^.

Ford *et al.*, Morais *et al.*, and Parra-Llorca *et al.* reported a higher abundance of Firmicutes at the phylum level^[33,35,37]^ in DHM-fed infants. Within the class Bacilli, two studies reported differences at the order level^[19,36]^, two studies reported differences at the family level^[32,35]^, and four studies reported differences at the genus level^[33-35,37]^. There are conflicting results at the order level, with Cong *et al.* stating a lower abundance of Lactobacillales and Bacillales at all three time points^[36]^, whereas Gregory *et al.* stated the abundance of the two orders to be higher in DHM-fed infants^[19]^. Within the reported outcomes in the class Bacilli, there are commonalities in the reported differences within the *Staphylococcaceae* family. Piñeiro-Ramos *et al.* reported higher abundances of the *Staphylococcaceae* family^[32]^, and in the same direction, Parra-Llorca *et al.*, Morais *et al.*, and Ford *et al.* showed an increased abundance of the *Staphylococcus* genus^[33,35,37]^.

Four studies stated differences at different taxonomic levels within the class Clostridia. Parra-Llorca *et al.* reported a higher abundance of the *Clostridiaceae* family^[35]^ in DHM-fed infants. Khumbare *et al.* and Parra-Llorca *et al.* stated a higher abundance of the genus *Clostridium*, whereas only Morais *et al.* stated a lower abundance in the DHM cohorts^[34-37]^.

#### Actinobacteria

Seven studies reported consistent differences in the Actinobacteria phylum at different taxonomic levels^[19,31,33-37]^. Ford *et al.*, Morais *et al.*, and Parra-Llorca *et al.* stated lower abundances of Actinobacteria at the phylum level in the DHM groups^[33,35,37]^. Cong *et al.* stated Actinomycetales at the order level have lower abundances^[36]^. There are quite some commonalities in the Bifidobacteriales order in that Cong *et al.* stated lower abundance of the Bifidobacteriales order, while Ford *et al.*, Morais *et al.*, and Parra-Llorca *et al.* stated lower abundance of the *Bifidobacterium* genus and Khumbare *et al.* stated lower prevalence of the *Bifidobacterium* genus in the samples from mostly DHM-fed infants^[33-35,37]^. Arboleya *et al.* focused solely on the Bifidobacterial population at the species level^[31]^. Although in both feeding groups, *B. breve* and *B. longum ssp. longum* were the species with the highest abundances during the study period, there were some differences in the abundances of other species. Notably, *B. animalis ssp lactis*, *B. bifidum*, and *B. dentium* were higher in the DHM group in terms of relative abundance. Only Gregory *et al.* stated no differences in the succession of Bifidobacteriales^[19]^.

#### Proteobacteria

Ford *et al.* reported an increasing relative abundance of Proteobacteria in all study subjects over time^[33]^. Five studies reported differences in the Proteobacteria phylum at different taxonomic levels, with conflicting results^[32,34-37]^. Morais *et al.* stated lower abundances of the Proteobacteria phylum^[37]^. In the Enterobacterales order, Cong *et al.* stated higher abundances of Enterobacterales^[36]^. Khumbare *et al.* and Parra-Llorca *et al.* stated lower abundances of an unclassified genus of the *Enterobacteriaceae* family^[34,35]^. In addition, Morais *et al.* stated a lower abundance of the *E. coli* species and *Serratia* genus, whereas Parra-Llorca *et al.* stated a higher abundance of *Serratia*^[35,37]^.

In the Pasteurellales order, a similar pattern of inconsistency is visible. Cong *et al.* stated a lower abundance of the Pasteurellales order^[36]^. Piñeiro-Ramos *et al.* stated a higher abundance of the *Pasteurellaceae* family^[32]^. At the genus level, Parra-Llorca *et al.* stated higher abundances of the *Aggregatibacter* genus and lower abundances of the *Haemophilus* genus^[35]^.

#### Bacteroidetes

Three studies reported consistent differences in the Bacteroidetes phylum at different taxonomic levels^[33,35,37]^. At the phylum level, Morais *et al.* stated a lower relative abundance of Bacteroidetes^[37]^. At the genus level, both Ford *et al.* and Parra-Llorca *et al.* stated a lower abundance of *Bacteroides*^[33,35]^.

### Other reported outcomes

#### Fecal metabolites

Arboleya *et al.* reported findings on fecal short-chain fatty acids (SCFA) content through gas chromatography, identifying them as biomarkers of microbiota metabolism. The reported SCFAs were acetate, propionate, and butyrate. At one month of life, concentrations of fecal propionate were higher in the DHM group (*P* < 0.05). Concentrations of acetate were lower in the DHM group (*P* < 0.05). Concentrations at the earlier time points showed no differences^[31]^.

Khumbare *et al.* used calprotectin levels in stool as a marker for gut inflammation and showed it was higher in the DHM group (*P* = 0.02)^[34]^.

In the study by Morais *et al.*, fecal alkaline phosphatase (ALP) activity was measured as a supposed potential biomarker for NEC. They stated no difference in ALP activity between the DHM and MOM groups^[37]^.

Parra-Llorca *et al.* observed no significant differences in estimated metabolic profiles of the gut microbiome using predictive functional profiling between the DHM and MOM groups^[35]^.

#### Clinical outcomes

Three studies in this review reported differences in health outcomes between the DHM and the MOM cohorts. Morais *et al.* did not observe differences in growth between the two groups on the 26th day of life^[37]^. However, Ford *et al.* showed growth velocity and body weight at a PMA of 36 weeks to be lower in the DHM group (both adjusted *P*-values < 0.01). In addition, they showed the incidence of the composite outcome of NEC, spontaneous intestinal perforation, sepsis, severe bronchopulmonary dysplasia, and death to be higher in the DHM group (adjusted *P*-values < 0.02)^[33]^. On the contrary, Piñeiro-Ramos *et al.* showed no statistically significant difference in NEC incidence between the groups^[32]^.

## DISCUSSION

In this systematic review, we observed differences in the fecal microbiome of preterm infants fed predominantly with DHM, compared to those predominantly fed with MOM. These differences manifest in both diversity and composition across various taxonomic levels and are reported across several time points, from as early as the second day of life to approximately 36 weeks PMA. In the following sections, we will discuss these findings in more detail.

Our review provides evidence indicating that preterm infants predominantly fed with DHM exhibit reduced alpha diversity in their gut microbiome compared to those fed predominantly with MOM. However, this evidence is solely documented within subgroup B, which encompasses studies incorporating formula feeding alongside MOM or DHM. Therefore, the inferred effect could also be related to the quantity of formula milk received, rather than the difference between MOM and DHM feeding. These findings align with existing evidence on the microbiome composition of infants fed PF *vs.* MOM, reporting higher alpha diversity in the latter cohorts^[39]^. The only study that described an increase in alpha diversity in the DHM group focused only on *Bifidobacterium* species, rather than assessing overall diversity^[31]^.

The included studies employed different metrics for alpha diversity evaluation, including the Shannon- or Chao1-indices and OTU richness. Several studies reported multiple measures for alpha diversity with conflicting outcomes. For example, Morais *et al.* showed a difference in the Chao1 index, but not in Shannon index findings, underscoring that the results on alpha diversity are dependent not only on the analyzed cohort, but also on the selected metric^[37]^. This discrepancy limits the comparability of published data.

Our review yields mostly consistent evidence for differences in beta diversity, with distinct clustering of the gut microbiome based on feeding type from the tenth day of life onwards. These differences are apparent in both subgroups A and B. The two studies assessing the effects of multiple demographic factors on the Bray-Curtis dissimilarity index, showed that feeding type contributed more to the observed difference than other factors tested, such as gestational age, birth weight, postnatal age, or antibiotic administration^[19,36]^. Contradicting results were present in the study by Morais *et al.*, which found no differences in beta diversity between the two feeding groups. This apparent incongruity might be due to the relatively small sample size (61 samples, of which 14 samples were from the DHM group) included for 16S rRNA sequencing compared to the other included studies^[37]^.

At the taxonomic level, there was large heterogeneity in reporting methods. While some studies reported outcomes at the phylum level, others reported at the order, family, genus and/or species levels. Despite this methodological heterogeneity, consistent evidence emerges regarding several compositional differences. DHM feeding appeared to be associated with an increased abundance of the *Staphylococcaceae* family, possibly attributable to elevated levels of the *Staphylococcus* genus, as well as the *Clostridiaceae* family, likely attributable to the increased abundance of the *Clostridium* genus.

Conversely, a decreased abundance was observed in the *Bifidobacterium* genus and the Bacteroidetes phylum in DHM-fed preterm infants compared to those fed predominantly MOM. In addition to a diminished overall abundance of *Bifidobacterium*, Arboleya *et al.* showed several differences in the relative abundances of various Bifidobacterial species present between both feeding groups, with differences starting to occur as early as the second day of life^[31]^.

The observed differences in the gut microbiota between both subgroups might be associated with different health outcomes through various (patho)physiological processes. While reduced alpha diversity levels have been linked to adverse health outcomes in adults, it is unknown if it pertains to preterm infants as well^[40]^. At the compositional level, the observed summarized differences in this systematic review have been linked to adverse health outcomes. An increased abundance of the *Staphylococcaceae* family or *Staphylococcus* genus has been linked to late-onset sepsis in preterm infants^[41,42]^. Similarly, increased abundances of different genera and species within the Clostridia order have been linked to NEC in preterm infants^[43-46]^. Moreover, reduced Bacteroidetes and *Bifidobacterium* abundance have also been associated with NEC^[47-51]^. Notably, these compositional differences are not only confined to short-term neonatal outcomes; the abundance of *Bifidobacterium*, *Staphylococcus*, and Bacteroidetes during the neonatal period has been linked to adverse neurodevelopmental outcomes at two years of corrected age^[24,52-54]^. In addition, term-born infants generally have higher abundances of Bacteroidetes and *Bifidobacterium* and lower abundances of *Staphylococcus* compared to preterm infants^[55]^. Therefore, the results of this systematic review may indicate greater deviance from a healthy full-term microbiome when infants are provided with DHM as compared to MOM.

However, recent studies show that not only microbiome composition, but also its metabolic activity and effects on the host’s immune system influence different health outcomes^[56,57]^. This is underscored by some studies included in this systematic review. One study highlights different concentrations of fecal SCFAs, metabolites linked to intestinal immune and endocrine responses, among different types of feeding^[31]^. Another study showed higher calprotectin levels, indicative of more intestinal inflammation in the DHM group^[34]^.

To the best of our knowledge, this is the first systematic review focusing solely on the difference in gut microbiome composition between infants predominantly fed MOM or DHM. One other systematic review on health outcomes between MOM- and DHM-exposed infants provides information on gut microbiome diversity only when it is mentioned in the included articles^[18]^. However, that review was designed to find health outcomes rather than microbiome outcomes, so key publications reporting only gut microbiota are not included. In addition, two other recent systematic reviews on the associations of feeding practices and the gut microbiome in preterm infants did not focus on DHM, resulting in the omission of relevant studies on this topic^[58,59]^. However, the findings in these reviews are coherent with our results.

Our review entails multiple strengths, including the categorization of studies involving infants exclusively human milk-fed and those with mixed feeding regimens. This stratification enabled us to mitigate possible bias by the potential effects caused by any consumption of PF. Furthermore, since both gestational age and chronological age may influence microbiota composition, we reported the PMA in Figures 2 and 3 for easier comparison when analyzing stool samples. However, this approach oversimplifies the potential impact of individual maturity on gut microbiome composition. Limitations of this review include the inability to provide a quantitative synthesis due to the considerable heterogeneity in reported outcome measures in the original studies. This includes variations in the use of different diversity indices, the use of different gut microbiome analysis techniques, and the preferred reporting styles for taxonomic outcomes. Additionally, most studies did not account for the mode of delivery which may have a confounding effect on microbiota composition. Several studies excluded infants with adverse neonatal outcomes such as mortality, NEC, and sepsis, possibly omitting infants with the most aberrant gut microbiome development, thus potentially biasing the results of this review. However, this can be seen as an advantage as well, where relatively well-doing preterm infants form a more homogeneous group with less external influences, so that the influence of milk type is better recognized.

The absence of randomized controlled trials (RCTs) comparing MOM to DHM is a significant limitation of reviews on this topic. The reliance on observational studies necessitates cautious interpretation of findings due to potential confounding variables. Future research should link health outcomes to gut microbiota changes attributable to feeding with DHM *vs.* MOM. In addition, the mechanisms through which MOM exerts its benefits to the infant should be investigated and used to improve existing DHM processing techniques to increase benefits. For instance, Holder pasteurization results in significant loss of bioactive compounds like lactoferrin, cytokines, and growth factors, which are vital for a healthy gut microbiome in infants^[60]^. Alternative pasteurization techniques such as high-temperature short-time pasteurization, high-pressure pasteurization, and Ultraviolet C may better preserve these compounds and thereby affect the microbiome in infants, although studies on this matter are sparse^[61]^. A novel concept of inoculation of DHM with bits of MOM shows promising *in-vitro* results in restoring the human milk microbiome^[62,63]^. However, *in-vivo* studies are needed to fully assess the effects of inoculated DHM on clinically important outcomes.

In conclusion, our review reveals several differences in gut microbiota development of preterm infants fed predominantly DHM *vs.* those fed predominantly MOM. The gut microbial composition shows distinct clustering based on feeding type, with the DHM group potentially displaying lower alpha diversity levels. At the compositional level, a DHM diet is associated with an increased abundance of the *Staphylococcaceae* and *Clostridiaceae* family and a lower abundance of Bacteroidetes and the *Bifidobacterium* genus. The differences persist throughout the follow-up period examined in the included studies. Importantly, these observed differences have been associated with adverse health outcomes both in the short and long term, such as an increased incidence of NEC, sepsis, and neurodevelopmental delay beyond infancy.

## DECLARATIONS

### Copyright

© The Author(s) 2024.
