# Supplementary material for: Impact of mother’s own milk vs. donor human milk on gut microbiota colonization in preterm infants: a systematic review
Source: Microbiome Res Rep. 2024 Nov 21;4(1):8. doi: 10.20517/mrr.2024.44 (PMC11977380; doi:10.20517/mrr.2024.44)
Supplement: Supplementary file 1 [file mrr-4-1-8-SupplementaryMaterials.zip › Supplementary Table 1.docx]

**Supplimentary Table 1. Gut microbiota analysis techniques**

| **Author, Year, Country [ref]** | **Gut microbiota assessment method** | **DNA extraction** | **16S rRNA region** | **Sequencing platform** | **Data analysis pipeline** | **Reference database** |
| --- | --- | --- | --- | --- | --- | --- |
| Arboleya, 2020, Spain * [31] | NGS | QIAmp DNA stool kit | 16S rRNA-23S rRNA ITS | MiSq-Illumina | QIIME2 | Updated version of bifidobacterial ITS database |
| Cong, 2017, USA [36] | NGS | MoBio Power Soil or PowerMag Soil DNA isolation kit | V4 region | MiSq-Illumina | QIIME | Greengenes |
| Ford, 2019, USA [33] | NGS | PowerSoil DNA isolation kits | V4 region | MiSq-Illumina | QIIME | Silva |
| Gregory, 2016, USA  [19] | NGS | (Kit from MoBio) | V3-V4 region | MiSq-Illumina | QIIME | Greengenes |
| Kumbhare, 2022, Canada [34] | NGS | Zymo Quick DNA Fecal Microbe Miniprep kit | V4 region | MiSq-Illumina | Dada2 | HITdb, V1.0 |
| Morais, 2021, Portugal [37] | NGS | NZI Tissue gDNA Isolation Kit | V3-V4 region | MiSq-Illumina | QIIME | Greengenes |
| Parra-Llorca, 2018, Spain [35] | NGS | MasterPure Complete DNA&RNA Purification Kit | V3-V4 region | MiSq-Illumina | QIIME | Greengenes |
| Pineiro Ramos, 2020, Spain [32] | NGS | MasterPure Complete DNA&RNA Purification Kit | V3-V4 region | MiSq-Illumina | QIIME | Greengenes |

NGS: next generation sequencing; qPCR: quantitative polymerase chain reaction;

* this study only measured species composition of the genus *Bifidobacterium*
