# Supplementary material for: Impact of mother’s own milk vs. donor human milk on gut microbiota colonization in preterm infants: a systematic review
Source: Microbiome Res Rep. 2024 Nov 21;4(1):8. doi: 10.20517/mrr.2024.44 (PMC11977380; doi:10.20517/mrr.2024.44)
Supplement: Supplementary file 1 [file mrr-4-1-8-SupplementaryMaterials.zip › Supplementary File 2 - Modified Newcastle-Ottawa Scale.doc]

**NEWCASTLE - OTTAWA QUALITY ASSESSMENT SCALE**

**COHORT STUDIES**

*Note: A study can be awarded a maximum of one star for each numbered item within the Selection and Outcome categories. A maximum of two stars can be given for Comparability*

**Selection**

1) Representativeness of the exposed cohort

a) truly representative of the average preterm infant in the community **🟑**

b) somewhat representative of the average preterm infant in the community **🟑**

c) selected group of users

d) no description of the derivation of the cohort

e) Low amount of participants (n<10)

2) Selection of the non exposed cohort

a) drawn from the same community as the exposed cohort **🟑**

b) drawn from a different source

c) no description of the derivation of the non exposed cohort

e) Low amount of participants (n<10)

3) Ascertainment of exposure

a) secure record **🟑**

b) structured interview **🟑**

c) written self report

d) no description

4) Demonstration that outcome of interest was not present at start of study

a) yes **🟑**

b) no

**Comparability**

1) Comparability of cohorts on the basis of the design or analysis

a) study controls for amount or fraction of type of milk (mothers own milk, donor human milk, preterm formula or other type of milk) consumed **🟑**

b) study controls for any additional factor, such as gestational age, site of admittance, mode of delivery, birth weight. **🟑**

**Outcome**

1) Assessment of outcome

a) independent blind assessment **🟑**

b) record linkage **🟑**

c) self report

d) no description

2) Was follow-up long enough for outcomes to occur

a) yes **🟑**

b) no

3) Adequacy of follow up of cohorts

a) complete follow up - all subjects accounted for **🟑**

b) subjects lost to follow up unlikely to introduce bias - > 70 %, and a description of those lost **🟑**

c) follow up rate < 70% and no description of those lost

d) no statement
